# Screening and Transcriptional Analysis of Polyketide Synthases and Non-ribosomal Peptide Synthetases in Bacterial Strains From Krubera–Voronja Cave

**DOI:** 10.3389/fmicb.2019.02149

**Published:** 2019-09-13

**Authors:** Dominykas Bukelskis, Daiva Dabkeviciene, Laima Lukoseviciute, Airidas Bucelis, Ignas Kriaučiūnas, Jolanta Lebedeva, Nomeda Kuisiene

**Affiliations:** ^1^Institute of Biosciences, Department of Microbiology and Biotechnology, Life Sciences Center, Vilnius University, Vilnius, Lithuania; ^2^Institute of Biosciences, Department of Biochemistry and Molecular Biology, Life Sciences Center, Vilnius University, Vilnius, Lithuania

**Keywords:** Krubera–Voronja Cave, polyketide synthase, non-ribosomal peptide synthetase, transcriptional, RT-qPCR

## Abstract

Identification of novel bioactive compounds represents an important field in modern biomedical research. Microorganisms of the underexplored environments, such as deserts, hot springs, oceans, and caves are highly promising candidates for screening such metabolites. Screening for biosynthetic genes is the most effective strategy to characterize bioactivity in a certain environment. However, knowledge is either scant or non-existent about the expression of the biosynthetic genes encoding for various bioactive compounds in the microorganisms from the caves. The aim of the current study was to screen for the genes of polyketide synthases and non-ribosomal peptide synthetases in Krubera–Voronja Cave (43.4184 N 40.3083 E, Western Caucasus) bacterial isolates as well as to evaluate the expression of these genes under laboratory conditions. In total, 91 bacterial strains isolated from the cave were screened for the presence of polyketide synthase and non-ribosomal peptide synthetase genes. Phenotypically inactive strains were the main focus (the test group) of our study, while the strains with the identified antibacterial activity served as the control group. Our PCR-based screening clearly showed that the majority of the strains harbored at least one biosynthetic gene. Prediction of the putative products allowed us to identify bioactive compounds with antibacterial, anticancer, antifungal, anti-inflammatory, antimycoplasmic, antiviral, insecticidal, and thrombolytic activity. For most polyketide synthases and non-ribosomal peptide synthetases, putative products could not be predicted; they are unknown. Qualitative transcriptional analysis did not show substantial differences between the test group and the control group of the strains. One to four biosynthetic genes were constitutively expressed in all the tested strains, irrespective of the group. Quantitative transcriptional analysis of the constitutively expressed biosynthetic genes demonstrated that the expression of a particular gene could be affected by both the amount of the nutrients in the culture medium and the growth phase.

## Introduction

Identification of novel compounds with antibacterial, antifungal, anticancer, antiviral, antidiabetic, antiprotozoal, and other bioactivities constitutes an important field of the modern biomedical research. Microorganisms are the main targets in this research field because of the high potential to produce these bioactive compounds. It is generally accepted that the search for these compounds in new species of microbes as well as in unculturable microorganisms represents the most promising possibility to identify novel compounds (Goodfellow and Fiedler, 2010; Monciardini et al., 2014). Therefore, microorganisms from the underexplored environments, such as deserts, hot springs, oceans, and caves are highly promising candidates for the screening of novel bioactive metabolites (Ghosh et al., 2017). It should be noted that the bioactive potential of the cave microorganisms is still poorly characterized. Usually, cultivation-based phenotypic analysis was carried out in order to evaluate the antifungal, anticancer, and most frequently, the antibacterial activity (Ghosh et al., 2017; Belyagoubi et al., 2018). In some cases, the chemical structure of the bioactive compounds was also determined (Herold et al., 2005; Stankovic et al., 2012; Jiang et al., 2015, 2018; Lamprinou et al., 2015; Axenov-Gribanov et al., 2016).

In the laboratories, the phenotypic investigation of the bioactive compounds is quite complicated due to few reasons: small amounts of these compounds; low concentration (below the detection limits); biosynthesis of these compounds can be inducible, and certain experimental conditions can be inappropriate for the induction; the kind of bioactivity is unknown *a priori*, and hence, it can be very complicated to choose appropriate test organisms or cells (Ghosh et al., 2017). These challenges can cause underestimation of the real potential of microorganisms. Therefore, screening for biosynthetic genes is a more effective strategy to characterize bioactivity in certain environments. However, the knowledge of the genetic potential is still scant for caves. To the best of our knowledge, only a few papers on the microorganisms and the bioactive compounds have dealt with the identification of biosynthetic genes in the cultivable strains (Hodges et al., 2012), genome mining for biosynthetic genes (Maciejewska et al., 2016; Adam et al., 2018; Gosse et al., 2019), and analysis of bioactivity using the metagenomic approach (Riquelme et al., 2017). Results of genome mining were correlated with the only metabolomics study by Gosse et al. (2019). Although phenotypic antimicrobial activity of the cave isolates was detected in a few studies (Maciejewska et al., 2016; Riquelme et al., 2017; Adam et al., 2018), the involvement of polyketide synthases (PKS) and non-ribosomal peptide synthetases (NRPS) for the activity was not investigated.

Krubera–Voronja Cave (43.4184 N 40.3083 E, Western Caucasus) is one of the deepest caves in the world. Antibacterial activity of the cave bacterial isolates was phenotypically characterized previously and was found to be lower than expected for such a deep cave (Klusaite et al., 2016). It could be that the screening system and/or conditions were inappropriate or these isolates do not indeed encode enzymes that are necessary for the synthesis of some bioactive compounds, particularly polyketides and non-ribosomal peptides. The aim of the current study was to perform a screening for the biosynthetic genes encoding PKS and NRPS in the Krubera–Voronja Cave isolates, and to determine if these genes are expressed under laboratory conditions.

## Materials and Methods

### Bacterial Strains

Bacterial strains were previously isolated in our laboratory from water and sediment samples collected in Krubera–Voronja Cave (Kieraite-Aleksandrova et al., 2015). Phenotypic antibacterial activity of these strains against the test strains of *Micrococcus luteus* Sar1, *Bacillus thuringiensis* TL8, *Escherichia coli* BL21(DE3), and *Pseudomonas* sp. VR1 was determined previously (Klusaite et al., 2016). For the current work, three groups of Krubera–Voronja Cave bacterial strains were chosen: 54 strains without phenotypic antibacterial activity (a test group), 27 strains with phenotypic antibacterial activity (a control group), and 10 randomly selected strains with unknown antibacterial activity. The list of strains and their characteristics is shown in Supplementary Table S1.

### DNA Extraction and BOX-PCR Genotyping

For genomic DNA extraction, bacterial strains were cultivated using the following microbiological media: Actinomycete Agar, Hickey-Tresner Agar, Starch Casein Nitrate Agar (Cheeptham et al., 2013), Tryptic Soy Agar (Merck Millipore, Darmstadt, Germany), and Difco^TM^ ISP Medium 4. Genomic DNA was extracted from fresh grown cultures using the GeneJET Genomic DNA Purification Kit (Thermo Fisher Scientific, Waltham, MA, United States). BOX-PCR was performed as described in Klusaite et al. (2016). Products of amplification were analyzed by electrophoresis using 1% agarose gel.

### Screening for PKS and NRPS Genes

PCR amplification of PKS and NRPS genes was performed using degenerate primers (Rajendran, 1999; Schirmer et al., 2005; Wawrik et al., 2005; Savic and Vasiljevic, 2006; Wood et al., 2007; Radjasa et al., 2008; Gontang et al., 2010; Abderrahmani et al., 2011; Palomo et al., 2013; Tambadou et al., 2014; Amos et al., 2015; Aleti et al., 2017) (Supplementary Table S2). Amplification was carried out in 50 μL of reaction mixture containing PCR buffer with (NH_4_)_2_SO_4_, 2 mM MgCl_2_, 0.2 mM each dNTP, 0.25 μM of each primer, 1.25 U recombinant *Taq*DNA Polymerase, and 10 ng of bacterial genomic DNA in an Eppendorf Mastercycler EP Gradient (Eppendorf, Hamburg, Germany). Amplification conditions for each primer pair were selected according to the references listed in Supplementary Table S2. PCR products were analyzed by electrophoresis using 1% agarose gel. PCR products of the correct size were purified using either the GeneJET PCR Purification Kit or the GeneJET Gel Extraction Kit (Thermo Fisher Scientific), and cloned into *E. coli* DH5α using the CloneJET PCR Cloning Kit (Thermo Fisher Scientific) according to the manufacturer’s instructions. Recombinant plasmids were isolated using the ZR Plasmid Miniprep-Classic Kit (Zymo Research Corp., Irvine, CA, United States). Inserts of the recombinant plasmids were sequenced at the DNA Sequencing Centre (Life Sciences Center, Vilnius University, Lithuania); obtained sequences were assembled, edited, and analyzed using the SEQMAN and SEQBUILDER components of LASERGENE 6 (DNASTAR) (DNASTAR Inc., Madison, WI, United States) as well as the BLAST tool^1^. PKS and NRPS sequences were deposited in the GenBank under the acc. No. MK461202-MK461209, MK506997-MK506998, and MK532964-MK532981. The prediction of the putative polyketides, produced by the identified PKS, was performed using a bioinformatics tool NaPDoS^2^ (Ziemert et al., 2012). Similarity >85% of the ketosynthase domains at the amino acid level were interpreted as an indication that the query domains may be associated with the production of the same or a similar compound as those produced by the reference pathway. For the prediction of the putative non-ribosomal peptides the BLAST tool was used.

### Identification of Bacterial Strains

Amplification of 16S rRNA genes was done according to Kuisiene et al. (2002). Cloning of 16S rRNA genes as well as sequence analysis were performed as described by Klusaite et al. (2016). 16S rRNA gene sequences were deposited in the GenBank under the acc. No. MK422459-MK422471.

### RNA Isolation

Bacterial cultures were grown in the 1.0x and 0.5x Tryptic Soy Broth (Merck Millipore, Darmstadt, Germany). For *Arthrobacter* sp., *Bacillus* sp., *Pseudomonas* sp., and *Rhodococcus* sp., inoculation (1% vol/vol) was performed using fresh bacterial cultures grown on Tryptic Soy Agar plates at 30°C. Inocula (OD_600_ = 2) were prepared in the respective sterile growth medium. Bacterial cultures were grown in an orbital shaker Multitron Standard (Infors AG, Bottmingen-Basel, Switzerland) at 30°C, 180 rpm. Growth of the culture was monitored by measuring optical density at 600 nm. For *Streptomyces* sp., the growth curve was constructed according to Manteca et al. (2005) using a diphenylamine colorimetric method (Zhao et al., 2013). Sampling of all the strains was performed in the middle of the exponential growth phase, at the transition phase, and at the beginning of the stationary growth phase. RNA isolation was done using the GeneJET RNA Purification Kit (Thermo Fisher Scientific) according to the manufacturer’s instructions. The integrity of RNA samples was analyzed using 1% agarose gel. RNA samples were stored at –20°C until use.

### Synthesis of cDNA

Total RNA samples were treated with dsDNase (Thermo Fisher Scientific) to remove any DNA contamination. In order to test for leftover DNA contamination in RNA samples, the samples were analyzed using 16S rRNA gene PCR (see above).

For reverse transcription PCR, the Maxima First Strand cDNA Synthesis Kit for RT-qPCR (Thermo Fisher Scientific) was used. The first-strand cDNA was synthesized using 1 μg of the DNA-free RNA template and random hexamer primers.

### Transcriptional Analysis

Both qualitative and quantitative PCR reactions were used for transcriptional analysis. The Primer-BLAST tool^3^ (Ye et al., 2012) was used for the construction of the qPCR primers targeting the PKS and NRPS sequences obtained in the current study. These primers were used for both qualitative and quantitative PCR reactions. The size of the expected PCR products was ≤200 bp, and melting temperature of the primers was in the range of 58–60°C.

PCR reaction mixture for qualitative transcriptional analysis was identical to that for screening of PKS and NRPS genes, except that 1 μL of cDNA was used instead of 10 ng of genomic DNA (see above). Qualitative PCR was conducted under the following conditions: initial denaturation at 95°C for 3 min followed by 30 cycles each consisting of 94°C for 30 s, 54°C for 30 s, and 72°C for 30 s, with a final extension step at 72°C for 5 min in an Eppendorf Mastercycler EP Gradient. PCR products were analyzed by electrophoresis, using 1% agarose gel.

The SensiFAST SYBR No-ROX Kit (Bioline, London, United Kingdom) was used for quantitative PCR (qPCR). Amplifications were performed on the CFX96 Touch^TM^ Real-Time PCR Detection System (Bio-Rad, Berkeley, CA, United States) in a standard two-step protocol according to the manufacturer’s instructions. SYBR Green dye containing PCR reaction mixtures with qPCR primers targeting PKS and NRPS genes were used for amplification and melt curve analysis. qPCR was carried out in 20 μL of reaction mixture containing 10 μL of 2x SensiFAST SYBR No-ROX Mix, 0.8 μL of each primer (10 μM), and 2 μL of cDNA template. Both undiluted and tenfold diluted cDNA served as the template in qPCR. qPCR was performed under the following conditions: polymerase activation at 95°C for 3 min followed by 40 cycles each consisting of 95°C for 5 s and 60°C for 30 s. The amplification specificity was confirmed by melt curve analysis. The thermal profile for melt curve determination started from an incubation at 65°C with a gradual increase in temperature (0.5°C/5 s) to 95°C. Absolute quantification approach was used in qPCR experiments. Calibration curves were built according to Le et al. (2014) with some modifications. Briefly, fragments of the genes were amplified using qPCR primers designed in the current study using genomic DNA as a template. PCR products were purified using the GeneJET PCR Purification Kit (Thermo Fisher Scientific), and tenfold dilution series were prepared to construct the calibration curves. The *R*^2^ values of all standard curves were >0.99. Results of amplifications were analyzed using the supporting CFX Manager software (v. 2.1) (Bio-Rad, Berkeley, CA, United States). Results were converted to the number of copies of a template using the calculator for determining the number of copies of a template^4^.

### Statistical Analysis

Sigma Plot 12.3 software (Systat Software Inc., San Jose, CA, United States) was used for statistical analysis. Multiple comparisons of gene expression levels were performed by *post hoc* tests following two-way ANOVA, evaluating the impact of two variables (the amount of the nutrients as well as the culture growth phase) on gene expression levels. The statistical tests were considered significant at *P* < 0.05.

## Results

### Screening for PKS and NRPS Genes

In total, 91 bacterial strains isolated from Krubera–Voronja Cave were screened for the presence of PKS and NRPS genes. The strains without phenotypic antibacterial activity were the main target of our study. For screening, we used both broad spectrum primers [for example, degenerate primers A3F/A7R, targeting NRPS adenylation domains (Ayuso-Sacido and Genilloud, 2005)] and specific primers for the detection of the biosynthetic genes of the particular compound [for example, degenerate primers Am1-F/Tm1-R, targeting adenylation and thiolation domains of mycosubtilin synthetase (Tapi et al., 2010)]. Before amplification of PKS and NRPS genes, all strains were subjected for BOX-PCR genotyping to be sure that all analyzed strains are different and do not represent re-isolation of the same strain.

One to seven PCR products of the correct size were obtained in the case of 72 strains (79.1% of all tested strains) (Supplementary Table S1). It should be noted that PKS and NRPS genes were detected in all three groups of the strains, although the percentage was higher for the randomly selected strains (90.0% of the tested strains) as well as for the phenotypically active strains (88.9% of the tested strains) than for the strains without phenotypic antibacterial activity (72.2% of the tested strains).

In order to determine PKS and NRPS gene sequences, PCR products of some of the strains were cloned and sequenced. The strains for which amplification was successful with a few different PCR primers have been chosen for this analysis. Results of these experiments are shown in Table 1. It should be noted that for all the strains, except *Streptomyces* sp. 1350R2-18, some of the sequenced clones were not PKS or NRPS gene fragments. We identified ABC transporters, peptidases, esterases, methyltransferases, dehydrogenases, cysteine desulfhydrases, sigma as well as anti-sigma factors, among others (data not shown). Some clones represented hypothetical proteins. Although we were not sure that some of these hypothetical proteins could be unidentified and unannotated PKS or NRPS genes, they were removed from further experiments for an accurate transcriptional analysis.

**TABLE 1:** The PKS and NRPS genes identified in this study.

**Strain (16S rRNA gene acc. No)**	**BLAST hit (similarity %)**	**PKS/NRPS acc. No.**
*Pseudomonas* sp. 230R1-NA30-2 (MK422468)	*Pseudomonas* sp. FDAARGOS_380, CP023969.1, 3940681-3941393, NRPS (95%)	MK532974 (NRPS)
	*Pseudomonas* sp. bs2935, LT629744.1, 2016802-2017295, NRPS domain TIGR01720 (94%)	MK532966 (NRPS)
*Streptomyces* sp. 1350R1-ISP30-2 (MK422469)	*Streptomyces* sp. ZFG47 plasmid, CP030074.1, 818619-817922, NRPS (82%)	MK532975 (NRPS)
	Uncultured bacterium clone C31_76, KC230134.1, putative type II PKS ketosynthase alpha subunit gene, partial cds (99%)	MK461206 (type II PKS)
	*Streptomyces* sp. 4F, CP013142.1, 304375-303895, 7743398-7743878, beta-ketoacyl synthase (94%)	MK461203 (type II PKS)
	*Micromonospora purpureochromogenes* strain DSM 43821, LT607410.1, 5028661-5028467, PKS (83%)	MK506997 (type I PKS)
	*Streptomyces platensis* subsp. *malvinus* strain NBRC 13827, clone plat13827ks-N3a, AB431103.1, 380-686, PKS, partial cds (83%)	MK461202 (type I PKS)
*Streptomyces* sp. 1350R2-18 (MK422462)	*Arthrobacter* sp. FB24, CP000454.1, 3474579-3474137, amino acid adenylation domain protein	–
	*Streptomyces* sp. strain MM21, KX708110.1, 625-1215, type II polyketide synthase 3, complete cds (92%)	MK461207 (type II PKS)
	*Streptomyces lunaelactis* strain MM109, CP026304.1, 6821677-6822155, beta-ACP synthase (90%)	MK461204 (type II PKS)
*Bacillus* sp. 1410WF1-ACT30-4 (MK422465)	*Bacillus subtilis* subsp. *subtilis* strain N3-1, CP032865.1, 2965817-2966238, NRPS (99%)	MK532964 (NRPS)
	*Bacillus subtilis* strain QB61, CP029461.1, 291662-292063, surfactin NRPS SrfAA (98%)	MK532967 (NRPS)
*Pseudomonas* sp. 1410F3-ACT20-6 (MK422463)	*Pseudomonas mandelii* strain LMG 21607, LT629796.1, 92574-92339, NRPS domain TIGR01720 (99%)	MK532969 (NRPS)
*Pseudomonas* sp. 1410F3-HT30-5 (MK422464)	*Pseudomonas mandelii* strain LMG 21607, LT629796.1, 84966-84734, NRPS domain TIGR01720 (97%)	MK532970 (NRPS)
*Pseudomonas* sp. 1410F3-ISP4-4 (MK422471)	*Rhizobacter gummiphilus* strain NBRC 109400, CP024645.1, 2406444-2407132, NRPS (87%)	MK532976 (NRPS)
	*Pseudomonas mandelii* JR-1, CP005960.1, 1688544-1688131, NRPS (99%)	MK532968 (NRPS)
	*Pseudomonas mandelii* strain LMG 21607, LT629796.1, 83994-83555, NRPS domain TIGR01720 (98%)	MK532965 (NRPS)
*Streptomyces* sp. 1410F3-ISP30-2 (MK422470)	*Streptomyces fungicidicus* strain TXX3120, CP023407.1, 2275827-2275447, NRPS (92%)	MK532977 (NRPS)
	*Streptomyces calvus* strain ATCC 13382, KT362217.1, 19116-18869, WS9326 biosynthetic gene cluster, complete sequence, NRPS (76%)	MK532971 (NRPS)
	Uncultured bacterium clone C31_76, KC230134.1, 1-573, putative type II PKS ketosynthase alpha subunit gene, partial cds (95%)	MK461208 (type II PKS)
	*Streptomyces* sp. SM18, CP029342.1, 96307-95799, beta-ketoacyl synthase (90%)	MK461205 (type II PKS)
	*Micromonospora* sp. strain GMKU326, LC021382.1, 19128-19372, *mak* gene cluster for maklamicin biosynthesis (83%)	MK506998 (type I PKS)
*Rhodococcus* sp. 1500R1-ACT30-2 (MK422459)	*Rhodococcus erythropolis* R138, CP007255.1, 5004067-5003360, putative NRPS (99%)	MK532978 (NRPS)
	*Rhodococcus erythropolis* R138, CP007255.1, 3781120-3781386, NRPS (97%)	MK532972 (NRPS)
*Pseudomonas* sp. 1550R3-HT30-5 (MK422466)	*Pseudomonas* sp. ACM7, CP024866.1, 752283-751613, NRPS (93%)	MK532979 (NRPS)
*Planomicrobium* sp. 1550R3-TSA4-4 (MK422467)	*Streptomyces cavourensis* strain 1AS2a, CP024957.1, 4117875-4118476, beta-ketoacyl synthase (99%)	MK461209 (type II PKS)
*Arthrobacter* sp. 1620R1-ACT30-4 (MK422460)	*Paenarthrobacter aurescens* TC1 plasmid pTC1, CP000475.1, 178531-177849, putative NRPS (74%)	MK532980 (NRPS)
*Pseudomonas* sp. 26TSA30-6A (MK422461)	*Pseudomonas* sp. ACM7, CP024866.1, 667574-666861, NRPS (94%)	MK532981 (NRPS)
	*Pseudomonas chlororaphis* subsp. *aureofaciens* strain ChPhzS23, CP027748.1, 4712737-4712469, pyoverdine chromophore precursor synthetase PvdL (87%)	MK532973 (NRPS)

In general, BLAST hits belonged to the same genus as the respective tested cave strain. The largest discrepancy was recorded for *Planomicrobium* sp. 1550R3-TSA4-4 (phylum *Firmicutes*) when the highest similarity with beta-ketoacyl synthase of *Streptomyces cavourensis* (phylum *Actinobacteria*) was determined. Database search could not find any PKS sequence of *Planomicrobium* sp., and this could be the reason of such discrepancy.

The extent of sequence similarity of the identified PKS and NRPS genes differed among the strains. For some genes, it was quite high – in the range from 90 to 99% (Table 1). But in some cases the sequence similarity was lower, and the lowest similarity (74%) was determined for one NRPS gene (MK532980) from *Arthrobacter* sp. 1620R1-ACT30-4. The highest PKS and NRPS gene similarity with the BLAST hits was identified for genera *Bacillus* (98–99%) and *Rhodococcus* (97–99%), while the lower similarity was determined for genera *Pseudomonas* (87–99%), *Streptomyces* (76–99%), and *Arthrobacter* (74%).

Sequence analysis using NaPDoS and BLAST tools was carried out in order to predict the putative products of the identified genes. For PKS, percentage identity >85% was obtained only for sequence MK461202 (*Streptomyces* sp. 1350R1-ISP30-2) – percentage identity with ketosynthase domain of PKS involved in biosynthesis of the spinosyn family of macrolides was 86%. Insecticidal activity of this family of polyketides has been previously reported (Bond et al., 2004; Kirst, 2010). Percentage identity for the other sequences was in the range of 40–81%, i.e., below the threshold that allows to reliably associate these sequences with the reference pathways. Consequently, the putative products of the other PKS were not predicted. For NRPS, only MK532967 (*Bacillus* sp. 1410WF1-ACT30-4) could be associated with the production of surfactin-like lipopeptides, the family of lipopetides with the previously reported diverse bioactivity (Wu et al., 2017). For the other NRPS, putative products could not be predicted.

### Qualitative Transcriptional Analysis

As previously mentioned, PKS and/or NRPS genes were detected not only in the phenotypically active strains, but also in the strains without antimicrobial activity. We wanted to find whether these genes are expressed under laboratory conditions in the latter strains. To answer this question, qualitative transcriptional analysis was carried out using six phenotypically inactive strains: *Streptomyces* sp. 1350R2-18, *Bacillus* sp. 1410WF1-ACT30-4, *Pseudomonas* sp. 1410F3-ACT20-6, *Pseudomonas* sp. 1410F3-HT30-5, *Pseudomonas* sp. 1410F3-ISP4-4, and *Streptomyces* sp. 1410F3-ISP30-2 (the test group). A few phenotypically active strains (*Pseudomonas* sp. 230R1-NA30-2, *Rhodococcus* sp. 1500R1-ACT30-2, *Arthrobacter* sp. 1620R1-ACT30-4, and *Pseudomonas* sp. 26TSA30-6A) as well as *Streptomyces* sp. strain 1350R1-ISP30-2 with unknown antibacterial activity were also included and served as the control group.

Impact of two parameters – microbial culture growth phase as well as the amount of nutrients in the culture medium – on gene expression levels was evaluated by transcriptional analysis. In order to determine impact of the first parameter, microbial cells were collected at three successive phases of the culture growth curve. To evaluate the second parameter, microbial cultures were cultivated in 1.0x (rich medium) and 0.5x (poor medium) Tryptic Soy Broth. As degenerate primers were used for screening of PKS and NRPS genes, there was amplification of unspecific templates in parallel with the target genes (see above), the specific primers targeting certain sequences were constructed for the transcriptional analysis (Table 2).

**TABLE 2:** Primers constructed in this study for transcriptional analysis.

**Strain**	**Target sequence**	**Forward primer (5′→3′)**	**Reverse primer (5′→3′)**	**Product size, bp**
*Pseudomonas* sp. 230R1-NA30-2	MK532974	Ju_16_NRPS_2F: CTCCGATGATGTGGCGTTCT	Ju_16_NRPS_2R: TACAAGCGGTTGTTGTCGGT	93
*Streptomyces* sp. 1350R1-ISP30-2	MK461202	L_14_PKS_4F: GTTGGTGGCGTTGCATCTG	L_14_PKS_4R: GAGACCATCACCGTCACACC	87
	MK461203	L_14_PKS-1F: CTGCAGAACCTCTGGAGCAA	L_14_PKS-1R: AGATTGACGGCGTAGAACCAG	77
	MK461206	L_14_PKS_3F: CTCGAGGAGCTCGAACACG	L_14_PKS_3R: TCAGCCCGGTCATGTGGTA	100
	MK506997	L_14_PKS_2F: CCGGCGTCATCAAAATGGTG	L_14_PKS_2R: CTGATGCCGAAGGAGGAGAC	181
	MK532975	L_14_NRPS_2F: CTCGTCAACGGATACGGACC	L_14_NRPS_2R: GTACGTACACACGGGTGCC	130
*Streptomyces* sp. 1350R2-18	MK461204	D_18_PKS-1F: TCATGACCCGACCAAATGGG	D_18_PKS-1R: TGCCCTCCTGAGTGGATTTC	131
	MK461207	D_18_PKS-2F: TCGATCAAGTCCATGGTGGG	D_18_PKS-2R: ACGTAGTCGAGGTCGCATTC	143
*Bacillus* sp. 1410WF1-ACT30-4	MK532964	I_4_NRPS_1F: CTGCTGCCTTTGTGACGATG	I_4_NRPS_1R: ATTTCTTCCTGCGGCGTTCT	130
	MK532967	I_4_NRPS_2F: GCGGATACCGGATTGAGCTT	I_4_NRPS_2R: ACGGCAAGCACAACACTTTC	82
*Pseudomonas* sp. 1410F3-ACT20-6	MK532969	I_13_NRPS_1F: GTCGCCTACATGCTCGAAGA	I_13_NRPS_1R: GATCACGTAGGCCAGGTTGT	180
*Pseudomonas* sp. 1410F3-HT30-5	MK532970	I_6_NRPS_1F: ATGATCGAAGACAGCGGCAT	I_6_NRPS_1R: GATCACGTAGGCGAGGTTGT	171
*Pseudomonas* sp. 1410F3-ISP4-4	MK532965	L_3_NRPS_1F: GGTTTCCGCATCGAATTGGG	L_3_NRPS_1R: TAGTCCGGCAGATCCACCTT	197
	MK532968	L_3_NRPS_2F: CTCAATGCCAACGGCAAACT	L_3_NRPS_2R: GCCCAGATATTCGCCAGTGT	122
*Streptomyces* sp. 1410F3-ISP30-2	MK461205	L_5_PKS_1F: CGAACTGCAACGGCTGTG	L_5_PKS_1R: TGTTGACGGCGTAGAACCAG	80
	MK461208	L_5_PKS_3F: TCCATCAAGTCCATGGTCGG	L_5_PKS_3R: CTGATGCCTGAGGGCCAG	84
	MK506998	L_5_PKS_2F: GCCGGAGTCATCAAGATGGT	L_5_PKS_2R: CACACCGAAGGAGGAGACAC	180
	MK532971	L_5_NRPS_1F: GAGATCGCCGAACTGACCC	L_5_NRPS_1R: GACCCGAGGTGTAGATGACG	94
	MK532977	L_5_NRPS_2F: GTGATCTACACGTCGGGGTC	L_5_NRPS_2R: GAGCGCGAAGTCCTCCAG	101
*Rhodococcus* sp. 1500R1-ACT30-2	MK532978	A_1_NRPS_2F: AGACCGCTACGACATTCGAC	A_1_NRPS_2R: GGTGCATTCGCAGTGAACAG	190
*Arthrobacter* sp. 1620R1-ACT30-4	MK532980	A_9_NRPS_1F: CAACGTTGTTGGCGACACTC	A_9_NRPS_1R: TGGGGCCGTACACATTCAAG	135
*Pseudomonas* sp. 26TSA30-6A	MK532973	A_16_NRPS_1F: GGAATACCCGCTGGATCGTT	A_16_NRPS_1R: CGTCCTCTTCGAGACACCAG	134
	MK532981	A_16_NRPS_2F: ATGACAGTTTGTGGACCCCG	A_16_NRPS_2R: ACCGCCAAAGCAATACACCT	155

**FIGURE 1:**
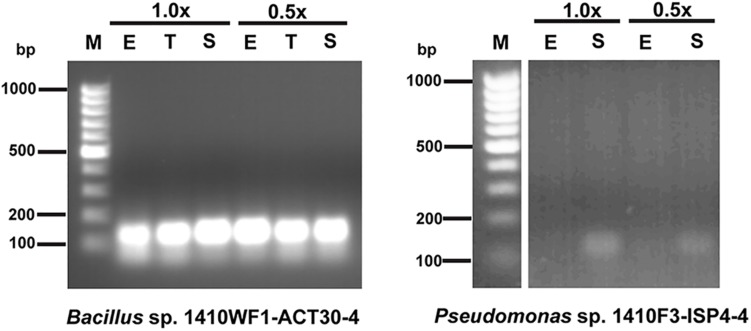
Qualitative transcriptional analysis of NRPS genes MK532964 in *Bacillus* sp. 1410WF1-ACT30-4 and MK532968 in *Pseudomonas* sp. 1410F3-ISP4-4. 1.0x, 1.0x Tryptic Soy Broth; 0.5x, 0.5x Tryptic Soy Broth; E, exponential phase; T, transition; S, stationary phase; M, Thermo Scientific GeneRuler 100 bp DNA Ladder (Thermo Fisher Scientific).

The examined NRPS genes were constitutively expressed in the *Bacillus* sp. 1410WF1-ACT30-4, *Pseudomonas* sp. 1410F3-ACT20-6, and *Pseudomonas* sp. 1410F3-HT30-5 and depended neither on the growth phase nor on the amount of nutrients. For the other two strains, the expression of different NRPS genes differed. Some genes were expressed constitutively (*Pseudomonas* sp. 1410F3-ISP4-4, MK532965; *Streptomyces* sp. 1410F3-ISP30-2, MK532971), while one was not expressed at all under experimental conditions (*Streptomyces* sp. 1410F3-ISP30-2, MK532977). Expression of MK532968 (strain *Pseudomonas* sp. 1410F3-ISP4-4) depended on the growth phase – it was not detected during the exponential growth phase in both 1.0x and 0.5x Tryptic Soy Broth. NRPS genes were constitutively expressed in the control group. Examples of constitutive and growth phase-dependent expression of NRPS genes are shown in Figure 1.

Expression of PKS genes was examined in three strains of *Streptomyces* sp.: 1350R2-18 and 1410F3-ISP30-2 from the test group, and 1350R1-ISP30-2 from the control group. In the test group, some genes (MK461207 and MK461204 from the strain 1350R2-18 as well as MK461208 and MK461205 from the strain 1410F3-ISP30-2) were constitutively expressed, while the expression of MK506998 (strain 1410F3-ISP30-2) was not detected. In the control strain, MK506997 was not expressed, while other genes (MK461206, MK461203, and MK461202) were expressed constitutively.

### Quantitative Transcriptional Analysis

**FIGURE 2:**
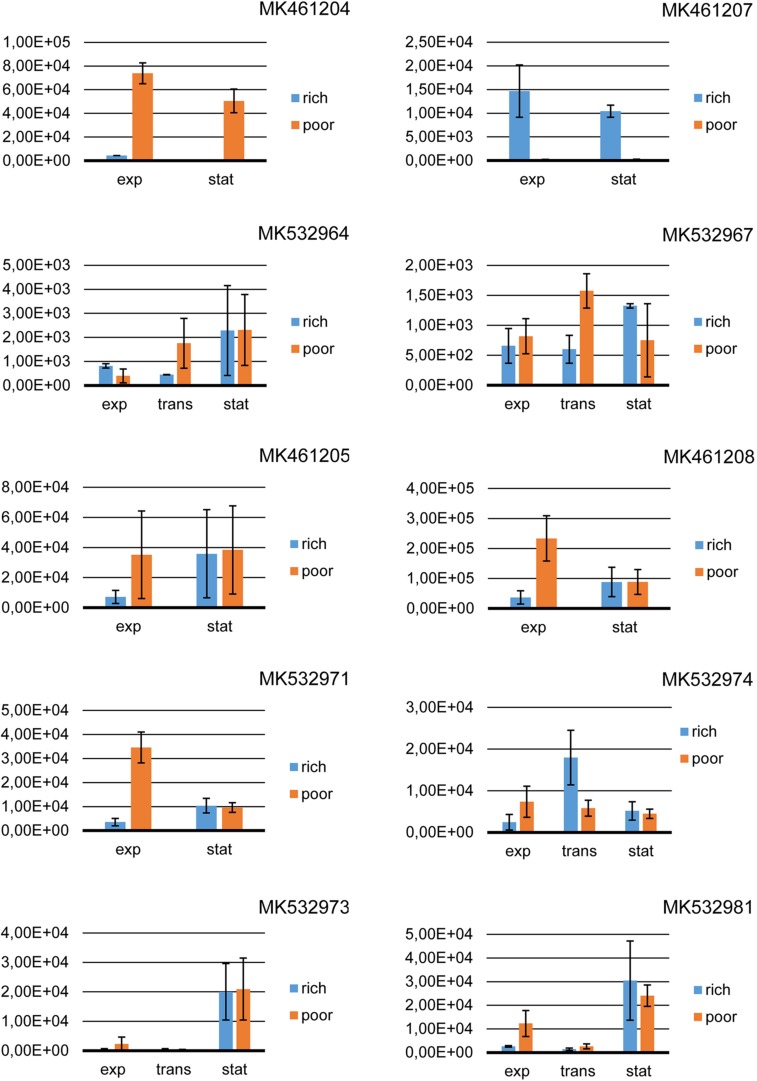
Quantitative reverse transcription PCR of PKS and NRPS genes. Rich, 1.0x Tryptic Soy Broth; poor, 0.5x Tryptic Soy Broth; exp, the exponential growth phase; trans, the transition phase; stat, the stationary growth phase.

Bacterial strains *Pseudomonas* sp. 230R1-NA30-2, *Streptomyces* sp. 1350R2-18, *Bacillus* sp. 1410WF1-ACT30-4, *Streptomyces* sp. 1410F3-ISP30-2, and *Pseudomonas* sp. 26TSA30-6A were chosen for quantitative transcriptional analysis. Both *Pseudomonas* sp. strains had antibacterial activity, while the other three strains were phenotypically inactive. At least two PKS and/or NRPS genes of these strains were constitutively expressed in the qualitative transcriptional analysis. The only exception was strain 230R1-NA30-2, with a single constitutively expressed NRPS gene MK532974. The constitutively expressed genes have been chosen for quantitative analysis in order to reveal the differences in their expression. Results of quantitative transcription experiments are shown in Figure 2.

In the strain *Streptomyces* sp. 1410F3-ISP30-2, the expression levels of both the PKS genes (MK461205 and MK461208) were affected neither by the growth phase (*P* = 0.563 and *P* = 0.409) nor by the amount of the nutrients (*P* = 0.580 and *P* = 0.124). In the case of NRPS gene MK532971, the growth medium had a significant impact on the expression level (*P* = 0.016). The number of the copies of a template tended to differ (*P* = 0.073) under the influence of the growth phase, and a significant impact of the growth phase was determined in the poor medium (*P* = 0.009) (Figure 2).

In *Streptomyces* sp. 1350R2-18, the expression of two PKS genes completely differed. Neither the growth phase (*P* = 0.759) nor the amount of the nutrients (*P* = 0.639) affected the expression of the gene MK461207. In the case of the gene MK461204, there was a significant (*P* < 0.001) influence of the culture medium on the expression level, while the growth phase had no significant impact (*P* = 0.11) on it.

In the strain *Bacillus* sp. 1410WF1-ACT30-4, the expression level of both MK532964 and MK532967 was not significantly affected by either the growth medium (*P* = 0.74 and *P* = 0.52, respectively) or the growth phase (*P* = 0.33 for the gene MK532964 and *P* = 0.56 for the gene MK532967).

In the control strain *Pseudomonas* sp. 230R1-NA30-2, neither the growth phase (*P* = 0.381) nor the amount of nutrients (*P* = 0.138) had a significant impact on the expression of the NRPS gene MK532974. In the other control strain – *Pseudomonas* sp. 26TSA30-6A – the growth medium did not significantly influence the expression level of both MK532973 (*P* = 0.86) and MK532981 (*P* = 0.812). However, the growth phase had a significant impact on the expression of both NRPS genes (*P* = 0.024 and *P* = 0.033, respectively), and the main difference (an increase in the expression) was determined between the transition and stationary phases (*P* = 0.043 for the gene MK532973 and *P* = 0.044 for the gene MK532981) (Figure 2).

## Discussion

Oligotrophic caves are considered to represent an excellent source of bioactive compounds. The diversity of microbiota in the caves is immense, and new species are continuously described. These new species may produce novel, formerly unknown compounds with different bioactivities (Monciardini et al., 2014; Ghosh et al., 2017). Moreover, the oligotrophic conditions lead to competition among the microbial species for food and the microorganisms produce various compounds against each other. The best-known example of the warfare in this competition is biosynthesis of antimicrobials including both narrow-spectrum peptides bacteriocins and broad-spectrum antimicrobials – polyketides and non-ribosomal peptides (Bauer et al., 2018). From this viewpoint, Krubera–Voronja Cave should be an excellent choice for the screening for bioactive compounds (Klusaite et al., 2016). However, in our previous work, the percentage of the strains with the phenotypic antimicrobial activity was lower than expected. Thus, we have tried to find the reason for the lower antimicrobial activity. We performed experiments to find if these strains encode the required biosynthetic genes, and/or to see if our experimental conditions were inappropriate for the detection of the bioactive compounds.

Phenotypically inactive strains were the main focus (the test group) of our current study, while the strains with identified antibacterial activity served as the control group. Our PCR-based screening clearly showed that the majority of the strains harbored at least one PKS and/or NRPS gene, irrespective of the group of strains. Other authors (Maciejewska et al., 2016; Adam et al., 2018) have also reported the presence of the multiple biosynthetic genes in the genomes of the phenotypically inactive strains. However, the absence of the respective biosynthetic genes in some strains of the control group was surprising. In our opinion, this phenomenon could be caused by two reasons: these strains could harbor divergent PKS/NRPS genes and, consequently, the primers used in our study were not suitable for the amplification of these genes or their phenotypic antibacterial activity could be caused by the volatile metabolites (Klusaite et al., 2016) or enzymes and not by polyketides or non-ribosomal peptides.

In most cases, we were unable to predict the putative products of PKS and NRPS, despite the high sequence similarity between the identified genes and those in the databases. It should be noted that in almost all cases, the genes from our study were most similar to those from the genome annotations that had no information on the characteristics of the compounds produced by the PKS and NRPS genes. Only two compounds were predicted in our study – polyketide of the spinosyn family of insecticides and surfactin-like lipopeptide with a previously reported antibacterial, anticancer, antifungal, anti-inflammatory, antimycoplasmic, antiviral, and thrombolytic activity (Wu et al., 2017). Enzymes responsible for the production of these two compounds were not previously reported from the caves (Hodges et al., 2012; Maciejewska et al., 2016; Riquelme et al., 2017; Adam et al., 2018). When the bacterial strains are used as the test organisms for a phenotype-based screening for bioactive compounds [as in our previous study (Klusaite et al., 2016)], only antibacterial but no other activities (such as insecticidal, anticancer, antifungal) can be detected. Consequently, the real bioactive potential of the cave microorganisms usually remains underestimated.

Our results also revealed one more potential reason of our inability to phenotypically detect bioactive strains that produce polyketides and/or non-ribosomal peptides. *Bacillus* sp. 1410WF1-ACT30-4 was subjected for analysis as one of the representatives of the test group. The genes MK532964 and MK532967 encoding NRPS were involved in the biosynthesis of the unknown non-ribosomal peptide and surfactin-like lipopeptide, respectively. Antibacterial activity of surfactin was previously reported (Wu et al., 2017), but it is low and for *Listeria monocytogenes*, for example, 125–1000 μg/mL of the compound (Sabaté and Audisio, 2013) is needed. Therefore, the phenotypic antibacterial activity of the strain 1410WF1-ACT30-4 could be under the detection limits of our experiments despite the constitutive expression of the respective gene.

Even with a few different test systems used, the compounds cannot be detected if the appropriate PKS and NRPS are not expressed and the compounds are not produced. Production of polyketides and non-ribosomal peptides starts from the transcription of the respective biosynthesis genes (McLean et al., 2019). Therefore, we carried out the transcriptional analysis in order to find if the identified genes are expressed at all, and, if yes, to see if the expression is affected by the amount of the nutrients in the culture medium and/or by the culture growth phase. The choice of the parameters was based on the assumptions that: (1) the shortage of nutrients triggers competition among microorganisms; (2) secondary metabolites, such as antimicrobials, are usually produced during the stationary growth phase of bacteria. Qualitative transcriptional analysis did not show substantial differences between the test group (phenotypically inactive strains) and the control group (the strains with the antibacterial activity). From 1 to 4 PKS and/or NRPS genes were constitutively expressed in all tested strains, irrespective of the group. Therefore, a closer examination of the constitutively expressed genes was performed using quantitative transcriptional analysis.

For most of the genes, the growth phase had no significant impact on the expression levels. The only exceptions were NRPS genes MK532973 and MK532981 (*Pseudomonas* sp. 26TSA30-6A) that showed higher expression level in the stationary growth phase, as well as NRPS gene MK532971 (*Streptomyces* sp. 1410F3-ISP30-2) that showed higher expression level in the exponential growth phase. There was a significant impact of the amount of nutrients only for two genes (MK532971 and MK461204 from strains of *Streptomyces* sp.), the remaining genes in our study were not affected by this parameter. It is generally accepted that the production of the secondary metabolites is linked to nutrient limitation in the stationary growth phase. It means that most of the genes in our study do not conform to this generally accepted rule. The same tendency was observed by Amos et al. (2017) in *Salinispora* sp. Thus, our results indicate that the nature of these “secondary” metabolites should be reconsidered.

Amos et al. (2017) previously reported that in most cases transcription is a good indicator of the compound production. Expression of PKS and NRPS genes is regulated by the complex network of regulators acting as either activators or repressors at the transcription level (van der Heul et al., 2018; Wei et al., 2018; McLean et al., 2019). Despite extensive studies on the global regulator BldA, the only tRNA necessary for the translation of the UUA codons (Hou et al., 2018; van der Heul et al., 2018), little is known about regulation at the pre- and post-transcriptional levels, overall (Wei et al., 2018). Čihák et al. (2017) previously noted that despite the expression of the respective genes, the biosynthesis of complex secondary metabolites may not occur. So, this phenomenon could also be the reason of the phenotypic inactivity of these strains, and this supposition should be tested for our strains in the future.

## Conclusion

In summary, our results clearly showed that the majority of the phenotypically inactive Krubera–Voronja Cave strains encode PKS and/or NRPS genes that could be responsible for the production of polyketides and non-ribosomal peptides with the various bioactivities. At least one of these genes was expressed constitutively in every studied strain. Our results showed that phenotype-based analysis of the cave strains led to underestimation of the real potential of bioactivity of various strains. Therefore, genome mining for PKS and NRPS genes in parallel with the transcriptional analysis of the identified genes would be the more effective strategy to analyze and exploit bioactivity of the bacterial strains found in the caves.

## Data Availability

The datasets generated for this study can be accessed from GenBank, MK422459-MK422471, MK461202-MK461209, MK506997-MK506998, and MK532964-MK532981.

## Author Contributions

DB, LL, and NK designed the experiments. DB, LL, AB, and IK performed the research and analyzed the data. DD performed the statistical analysis. JL performed the sequence analysis *in silico*. NK directed the research, analyzed the data, and wrote the manuscript.

## Conflict of Interest Statement

The authors declare that the research was conducted in the absence of any commercial or financial relationships that could be construed as a potential conflict of interest.

## Footnotes

1 https://blast.ncbi.nlm.nih.gov/Blast.cgi

2 http://npdomainseeker.sdsc.edu//napdos_home.html

3 https://www.ncbi.nlm.nih.gov/tools/primer-blast/index.cgi?LINK_LOC=BlastHome

4 https://cels.uri.edu/gsc/cndna.html
