# Supplementary material for: Screening and Transcriptional Analysis of Polyketide Synthases and Non-ribosomal Peptide Synthetases in Bacterial Strains From Krubera–Voronja Cave
Source: Front Microbiol. 2019 Sep 13;10:2149. doi: 10.3389/fmicb.2019.02149 (PMC6753585; doi:10.3389/fmicb.2019.02149)
Supplement: Supplementary file 1 [file Data_Sheet_1.PDF]

TABLE S1. Characteristics of the strains used in this study.

Strain	Depth of isolation (m)	Rarely/frequently visited location	Phenotypic antibacterial activity	Number of PKS and NRPS PCR products of the correct size
220F3-ACT30-2	220	frequently	ND	none
230R1-ACT30-6	230	rarely	ND	PKS-PCR – 2; NRPS-PCR - 0
230R1-TSA4-1B	230	rarely	ND	PKS-PCR – 2; NRPS-PCR - 0
230R1-TSA4-1G	230	rarely	ND	PKS-PCR – 1; NRPS-PCR - 0
230R1-TSA4-1BG	230	rarely	ND	PKS-PCR – 1; NRPS-PCR - 0
230R3-ISP30-2	230	rarely	ND	PKS-PCR – 1; NRPS-PCR - 0
230R3-TSA30-8	230	rarely	ND	PKS-PCR – 1; NRPS-PCR - 0
1350R1-ISP30-2	1350	rarely	ND	PKS-PCR – 5; NRPS-PCR - 2
1410WF1-SCNA30-25	1410	frequently	ND	PKS-PCR – 2; NRPS-PCR - 2
14ISP4-5	ND	ND	ND	PKS-PCR – 4; NRPS-PCR - 2
230R3-SCNA30-1	230	rarely	negative	PKS-PCR – 1; NRPS-PCR - 3
230R3-SCNA30-3	230	rarely	negative	PKS-PCR – 3; NRPS-PCR - 3
500WF1-HT30-1	500	frequently	negative	PKS-PCR – 0; NRPS-PCR - 1
1215WF1-25	1215	frequently	negative	none
1215WF1-27	1215	frequently	negative	PKS-PCR – 1; NRPS-PCR - 0
1215F2-HT30-5	1215	frequently	negative	PKS-PCR – 0; NRPS-PCR - 1
1350R3-7	1350	rarely	negative	none
1350R3-11	1350	rarely	negative	none
1350R3-12	1350	rarely	negative	none
1350R2-13	1350	rarely	negative	PKS-PCR – 1; NRPS-PCR - 0
1350R2-3'	1350	rarely	negative	PKS-PCR – 1; NRPS-PCR - 0
1350R2-14	1350	rarely	negative	PKS-PCR – 0; NRPS-PCR - 1
1350R2-14'	1350	rarely	negative	none
1350R2-15	1350	rarely	negative	none
1350R2-16	1350	rarely	negative	none
1350R2-16'	1350	rarely	negative	none
1350R2-17'	1350	rarely	negative	none
1350R2-18	1350	rarely	negative	PKS-PCR – 2; NRPS-PCR - 1
1350R2-19	1350	rarely	negative	none

1350R2-20	1350	rarely	negative	none
1350R2-21	1350	rarely	negative	none
1350R2-35	1350	rarely	negative	none
1350R3-40	1350	rarely	negative	none
1350R1-ISP4-1	1350	rarely	negative	PKS-PCR – 0; NRPS-PCR - 1
1350R1-ISP30-1	1350	rarely	negative	PKS-PCR – 2; NRPS-PCR - 3
1410WF1-ACT30-4	1410	frequently	negative	PKS-PCR – 0; NRPS-PCR - 4
1410WF1-ACT30-6	1410	frequently	negative	none
1410WF1-SCNA30-1	1410	frequently	negative	PKS-PCR – 2; NRPS-PCR - 2
1410WF1-SCNA30-4	1410	frequently	negative	PKS-PCR – 1; NRPS-PCR - 3
1410WF1-SCNA30-5	1410	frequently	negative	PKS-PCR – 2; NRPS-PCR - 3
1410F3-ACT30-2	1410	frequently	negative	PKS-PCR – 1; NRPS-PCR - 2
1410F3-ACT20-6	1410	frequently	negative	PKS-PCR – 3; NRPS-PCR - 2
1410F3-HT30-2	1410	frequently	negative	PKS-PCR – 2; NRPS-PCR - 0
1410F3-HT30-5	1410	frequently	negative	PKS-PCR – 1; NRPS-PCR - 2
1410F3-ISP4-4	1410	frequently	negative	PKS-PCR – 2; NRPS-PCR - 4
1410F3-ISP30-1	1410	frequently	negative	PKS-PCR – 0; NRPS-PCR - 3
1410F3-ISP30-2	1410	frequently	negative	PKS-PCR – 3; NRPS-PCR - 3
1410F3-ISP30-3	1410	frequently	negative	PKS-PCR – 3; NRPS-PCR - 3
1500R1-HT30-12	1500	rarely	negative	PKS-PCR – 1; NRPS-PCR - 0
1500R1-TSA4-4	1500	rarely	negative	PKS-PCR – 1; NRPS-PCR - 0
1500R1-TSA4-5	1500	rarely	negative	PKS-PCR – 1; NRPS-PCR - 0
1550R1-ISP30-1A	1550	rarely	negative	PKS-PCR – 1; NRPS-PCR - 0
1550R1-TSA4-1	1550	rarely	negative	PKS-PCR – 2; NRPS-PCR - 0
1550R2-HT30-2	1550	rarely	negative	PKS-PCR – 2; NRPS-PCR - 0
1550R3-TSA4-4	1550	rarely	negative	PKS-PCR – 3; NRPS-PCR - 0
1620R1-SCNA30-5	1620	rarely	negative	PKS-PCR – 1; NRPS-PCR - 0
1640WF3-HT30-8	1640	frequently	negative	PKS-PCR – 1; NRPS-PCR - 1
1640F1-ACT30-5	1640	frequently	negative	PKS-PCR – 0; NRPS-PCR - 2
14ISP4-2	ND	ND	negative	PKS-PCR – 1; NRPS-PCR - 5
14ISP4-4	ND	ND	negative	PKS-PCR – 3; NRPS-PCR - 3
14ISP4-7	ND	ND	negative	PKS-PCR – 2; NRPS-PCR - 2
14SCNA30-2	ND	ND	negative	PKS-PCR – 1; NRPS-PCR - 1
14SCNA30-4	ND	ND	negative	PKS-PCR – 1; NRPS-PCR - 3

14SCNA30-6	ND	ND	negative	PKS-PCR – 0; NRPS-PCR - 1
220F2-TSA30-3	220	frequently	positive	PKS-PCR – 1; NRPS-PCR - 0
220F4-ACT30-1	220	frequently	positive	PKS-PCR – 0; NRPS-PCR - 2
220F4-ACT30-6	220	frequently	positive	PKS-PCR – 0; NRPS-PCR - 2
220F4-HT30-1	220	frequently	positive	PKS-PCR – 1; NRPS-PCR - 1
220F4-HT30-6	220	frequently	positive	PKS-PCR – 1; NRPS-PCR - 0
220F4-HT30-7	220	frequently	positive	PKS-PCR – 1; NRPS-PCR - 0
230R1-NA30-2	230	rarely	positive	PKS-PCR – 1; NRPS-PCR - 2
1215F2-ACT30-2	1215	frequently	positive	PKS-PCR – 1; NRPS-PCR - 2
1215F2-ACT30-4	1215	frequently	positive	PKS-PCR – 1; NRPS-PCR - 2
1350R3-ACT30-7	1350	rarely	positive	none
1500R1-ACT30-2	1500	rarely	positive	PKS-PCR – 3; NRPS-PCR - 3
1500R1-ACT30-3A	1500	rarely	positive	PKS-PCR – 1; NRPS-PCR - 1
1500R1-ACT30-4A	1500	rarely	positive	none
1500R1-ACT30-5A	1500	rarely	positive	PKS-PCR – 0; NRPS-PCR - 1
1500R1-ACT30-6A	1500	rarely	positive	PKS-PCR – 1; NRPS-PCR - 2
1550R3-HT30-3A	1550	rarely	positive	PKS-PCR – 1; NRPS-PCR - 2
1550R3-HT30-5	1550	rarely	positive	PKS-PCR – 2; NRPS-PCR - 1
1550R3-HT30-6A	1550	rarely	positive	none
1620R1-ACT30-1	1620	rarely	positive	PKS-PCR – 0; NRPS-PCR - 4
1620R1-ACT30-2A	1620	rarely	positive	PKS-PCR – 0; NRPS-PCR - 2
1620R1-ACT30-4	1620	rarely	positive	PKS-PCR – 1; NRPS-PCR - 3
1640F1-HT30-4A	1640	frequently	positive	PKS-PCR – 0; NRPS-PCR - 1
15ISP30-4	ND	ND	positive	PKS-PCR – 0; NRPS-PCR - 1
26ACT30-10	ND	ND	positive	PKS-PCR – 1; NRPS-PCR - 2
26TSA30-6A	ND	ND	positive	PKS-PCR – 2; NRPS-PCR - 4
26TSA30-7A	ND	ND	positive	PKS-PCR – 2; NRPS-PCR - 3
28TSA4-2	ND	ND	positive	PKS-PCR – 1; NRPS-PCR - 1

ND – no data.
