# Supplementary material for: Screening and Transcriptional Analysis of Polyketide Synthases and Non-ribosomal Peptide Synthetases in Bacterial Strains From Krubera–Voronja Cave
Source: Front Microbiol. 2019 Sep 13;10:2149. doi: 10.3389/fmicb.2019.02149 (PMC6753585; doi:10.3389/fmicb.2019.02149)
Supplement: Supplementary file 2 [file Data_Sheet_2.PDF]

TABLE S2. Primers used for polyketide synthase and nonribosomal peptide synthetase screening.

Primer	Sequence (5'→3')	Reference
**Nonribosomal peptide synthetase:**		
A2gamF/A3gamR	A2gamF: AAGGCNGGCGSBGCSTAYSTGCC	Tambadou et al., 2014
	A3gamR: TTGGGBIKBCCGGTSGINCCSGAGGTG	
A3F/A7R	A3F: GCSTACSYSATSTACACSTCSGG	Ayuso-Sacido and Genilloud, 2005
	A7R: SASGTCVCCSGTSCGGTAS	
Abl1-F/Tbl1-R	Abl1-F: GATSAWCARGTGAAAATYCG	Abderrahmani et al., 2011
	Tbl1-R: ATCGAATSKCCGCCRARATCRAA	
Af2-F/Tf1-R	Af2-F: GAATAYMTCGGMCGTMTKGA	Tapi et al., 2010
	Tf1-R: GCTTTWADKGAATSBCCGCC	
Am1-F/Tm1-R	Am1-F: CAKCARGTSAAAATYCGMGG	Tapi et al., 2010
	Tm1-R: CCDASATCAAARAADTTATC	
Ap1-F/Tp1-R	Ap1-F: AGMCAGCKSGCMASATCMCC	Tapi et al., 2010
	Tp1-R: GCKATWWTGAARRCCGGCGG	
As1-F/Ts2-R	As1-F: CGCGGMTACCGVATYGAGC	Tapi et al., 2010
	Ts2-R: ATBCCTTTBTWDGAATGTCCGCC	
degNRPS.i	degNRPS-1F.i: AARDSIGGIGSIGSITAYBICC	Schirmer et al., 2005
	degNRPS-4R.i: CKRWAICCICKIAIYTTIAYYTG	
Foxy/Roxy	Foxy: CTGGTCGGCAACCTGATGGAC	Wood et al., 2007
	Roxy: CAGGTACCGGATCAGCTCGTC	
MT F/R	MTF: CCNCGDATYTTNACYTG	Tambadou et al., 2014
	MTR: GCNGGYGGYGCNTAYGTNCC	
NRPS F1/F2/R	NRPS-F1: CGCTGACCCCCAACGGNAARBTNYA	Amos et al., 2015
	NRPS-F2: CGCGCGCATGTACTGGACNGGNGAYYT	
	NRPS-R: GGAGTGGCCGCCCARNYBRAARAA	
TGD/LGG	TGD: TACCGIACIGGIGATCTIGTICG	Rajendran, 1999
	LGG: ATIGAGTCICCICCIGGGIAAAAGAA	
**Polyketide synthase:**		
540F/1100R	540F: GGITGCACSTCIGGIMTSGAC	Wawrik et al., 2005
	1100R: CCGATSGCICCSAGIGAGTG	
ANSA F/R	ANSA-F: CCSGCSTTCACSTTCATCTC	Wood et al., 2007
	ANSA-R: AISYGGAICATIGCCATGTAG	
ARO-PKS F/R	ARO-PKS-F: GGCAGCGGITTCGGCGGITTCCAG	Wood et al., 2007
	ARO-PKS-R: CGITGTTIACIGCGTAGAACCAGGCG	

degKS.i	degKS2F.i: GCIATGGAYCCICARCARMGIVT<br>degKS5R.i: GTICCIGTICCRTGISCYTCIAC	Schirmer et al., 2005
EdyA/EdyE	EdyA: CCCCGCVCACATCACSGSCCTCGCSGTGAACATGCT<br>EdyE: GCAGGCKCCGTCSACSGTGTABCCGCCGCC	Gontang et al., 2010
K1F/M6R	K1F: TSAAGTCSAACATCGGBCA<br>M6R: CGCAGGTTSCSGTACCAGTA	Ayuso-Sacido and Genilloud, 2005
KS F/R	KS-F: CCSCAGSAGCGCSTSYTSCTSGA<br>KS-R: GTSCCSGTSCCGTGSGYSTCSA	Gontang et al., 2010
KSα F/R	KSαF: TSGCSTGCTTGGAYGCSATC<br>KSαR: TGGAANCCGCCGAABCCGCT	Hodges et al., 2012
KSαF_MicroKoc/KSβR	KSαF_MicroKoc: TSGRCTACRTCAACGCSCACGG<br>KSβR: TACSAGTCSWTCGCCTGGTTC	Palomo et al., 2013
KSDPQQF/KSHGTGR	KSDPQQF: MGNGARGCNNWNSMNATGGAYCCNCARCANMG<br>KSHGTGR: GGRTCNCCNARNSWNGTNCCNGTNCCRTG	Radjasa et al., 2008
MAK1/MAK3	MAK1: GACACSGCSTGYTCBTCGTCG<br>MAK3: CCGTTSGACGCRCCGTCCTGGTTSCA	Savic and Vasiljevic, 2006
PKS1 F/R	PKS1-F: CGGGGCACCGCCATSAACMASGRC<br>PKS1-R: SGCCCAGCGGGGTGSCSGTNCCGTG	Le et al., 2014
PKS2 F/R	PKS2-F: CCACCCGCTACGSSBHCCACMGT<br>PKS2-R: TCTGCTTGGTGCCGSWNCCGTGSGC	Le et al., 2014
Pks firmi_F/R	Pks firmi_F: GCNGGNCAYWSNYTNGGNGARTAYA<br>Pks firmi_R: CATRWANCKNSWRTGRAANGCNCC	Aleti et al., 2017
PO1/PO3	PO1: GCNTGTMGCTNTTYCCNGGNGG<br>PO3: CTGTGSCGSACYAGBAGCAGC	Savic and Vasiljevic, 2006
PS1/PS2	PS1: GGNACNCCNMANGGNGAMCC<br>PS2: CGNYGGAANSGGTANGTNGG	Savic and Vasiljevic, 2006
